# Efficacy and Safety of Bedside Removal of Tunnelled Hemodialysis Catheter by Noninterventional Nephrologists among Adult Patients in the King Abdulaziz University Hospital Hemodialysis Centre in Jeddah: A Retrospective Cohort Study

**DOI:** 10.1155/2023/6905528

**Published:** 2023-03-27

**Authors:** Abdullah Kashgary, Razan A. Almuhyawi, Reem R. Alhijri, Aseel M. Ba Durayq, Wed B. Alnagrani, Arwa J. Alharbi, Hamidah M. Al Khalaf, Haya S. Obaid, Ahmed Zaky Fadel, Mostafa Abdelsalam

**Affiliations:** ^1^Department of Medicine, Faculty of Medicine, King Abdulaziz University, King Abdulaziz University Hospital, Jeddah, Saudi Arabia; ^2^Faculty of Medicine, King Abdulaziz University, Jeddah, Saudi Arabia; ^3^Mansoura Nephrology and Dialysis Unit, Faculty of Medicine, Mansoura University, Mansoura, Egypt

## Abstract

This study aimed to assess the efficacy and safety of bedside removal of tunnelled hemodialysis catheter (TDC) by noninterventional nephrologists among adult patients. It is a retrospective study that involved 53 patients from March 2020 to February 2022 at the King Abdulaziz University Hospital (KAUH) Hemodialysis Centre in Jeddah, Saudi Arabia. Of the 53 participants, 60.4% were male and 40.6% female, and their mean age was 50.94 ± 18.89 years. The most common comorbidities were hypertension (HTN) in 47 (88.7%), diabetes mellitus (DM) in 24 (45.3%), and DM and HTN together in 23 (43.4%) patients. The most common site of TDC removal was the right internal jugular vein (77.4%). In 84.9% of the cases, the TDC was removed as an inpatient procedure, and in the majority of the cases (64.2%), the TDC was removed by a noninterventional nephrologist. The most common reasons for TDC removal were sepsis or clinical concerns for infection (64.2%) and TDC not needed (20.8%) due to recovery of the renal function or access maturation. Most patients (96.2%) suffered no complications; only one of 34 (%) patients with catheter removal by a noninterventional nephrologist had bleeding, which required more observation and monitoring before discharge on the same day. Our study revealed that the bedside TDC removal was well tolerated with a minimal complication rate.

## 1. Introduction

Tunnelled hemodialysis catheters (TDCs) were first created in 1987 and have since played a critical role in managing and treating hemodialysis patients [1]. In addition, TDCs for hemodialysis have become an essential part of treatment strategies for patients with end-stage renal disease (ESRD) [2]. Indications for using TDCs are older age, patients with comorbid conditions, insufficient planning before starting hemodialysis, delay in choosing appropriate treatment modality, scheduled living-donor transplantation, and needle phobia [3].

According to the recently released data, over 80% of the dialysis patients in the United States begin hemodialysis with a TDC. Infection, poor catheter function, discontinuation of dialysis, stenosis of the central veins, and device failure leading to lower blood flow rates and limited functional survival life of the patient are the main reasons for TDC removal [4, 5]. TDCs can be removed under various settings and by different healthcare professionals, including surgeons, interventional radiologists, interventional nephrologists, and noninterventional nephrologists at the bedside [5].

The length of time with a TDC is the most significant cumulative risk factor for catheter-associated bloodstream infections; hence any delay in TDC removal could harm patients. Quick hardware removal is crucial to avoid these issues, as it facilitates timely patient care [2, 5]. In Saudi Arabia, there has been an increase in the prevalence of ESRD, resulting in the greater use of TDCs [6]. Unfortunately, many hospitals need TDCs to be removed by a vascular surgeon or an interventional radiologist, which often delays patient care and leads to inefficient resource utilization [5].

A noninterventional nephrologist could perform TDC removal to alleviate the strain on healthcare resources. In addition, TDC removal at the bedside by a noninterventional nephrologist has previously been shown to be safe [2]. It is, therefore, vital to determine whether TDC can be withdrawn at the bedside in Saudi Arabia with the same level of care and safety.

This study aimed to assess the efficacy and safety of bedside removal of TDCs by noninterventional nephrologists comparing to other providers among adult patients at the KAUH Hemodialysis Centre in Jeddah, Saudi Arabia.

## 2. Participants and Methods

This retrospective study involved 53 patients from March 2020 to February 2022 at the KAUH Centre in Jeddah, Saudi Arabia. Adult patients (above 18 years old) undergoing hemodialysis who had TDC removed were included in the study. We used a predesigned checklist for recording patients' demographics like age (in years) and sex. Additionally, information regarding the type of healthcare provider (nephrologist or an intervention radiologist/nephrologist or surgeon), location of TDC removal (bedside or operation theatres), underlying medical conditions (diabetes mellitus (DM), dyslipidaemia, hypertension (HTN), or renal transplant), biochemical parameters (blood urea nitrogen (BUN), creatinine (Cr), platelet count (PLT), prothrombin time (PT), international normalised ratio (INR), white blood cell count (WBC), and hemoglobin (Hb) level were collected. Data were also obtained regarding any immediate complications (bleeding, hypotension, loss of consciousness, death, hospitalization related to catheter removal, and cardiac arrest). Based on convenience sampling, data were collected from the electronic medical records of the patients from the KAUH Centre, including all current patients.

The exclusion criteria were patients with absolute contraindications to TDC insertion, including age <18 years, active or current sepsis/bacteraemia, and uncontrolled coagulopathies, and patients with new-onset cardiorespiratory instability or with a history of central vein stenotic/occlusive disease.

The inclusion criteria were adult patients who underwent TDC placement at the bedside utilizing anatomic landmarks and ultrasound guidance was used with all of the patients.

Outcomes considered to evaluate the safety of bedside TDC insertion included the incidence of procedural complications such as bleeding, arterial puncture, venous air embolism, arrhythmias, pneumothorax, hemothorax, and catheter tip malposition.

The data were analyzed using the SPSS program version 26. The Shapiro–Wilk test was used to test the normality of variables. The parametric variables were expressed as mean ± SD, while nonparametric variables were expressed as median (minimum-maximum). Qualitative variables were described as number (%). For comparison between the 3 groups, one-way analysis of variance (ANOVA) and Kruskall–Wallis test were used for parametric and nonparametric variables, respectively. The Chi-squared test (*χ*2) was performed to assess the association between qualitative data reported as numbers and percentages. The Monte Carlo test was used as a correction of the Chi-square test when more than 25% of cells have count less than 5 in tables (>2 *∗* 2). A *p* value of less than 0.05 was considered statistically significant.

Ethical approval for the study was obtained from the King Abdulaziz University, the Faculty of Medicine Research Ethics Committee. As our study involved a retrospective review of medical records, no patient participation was required. All data collected were reported in an aggregated and anonymized format.

## 3. Results

In our study, of the 53 participants, 60.4% were male and 40.6% female. The mean age of the patients was 50.94 ± 18.89 years. Moreover, the most common comorbidities were hypertension (HTN) in 47 (88.7%), diabetes mellitus (DM) in 24 (45.3%; including 5 (9.3%) patients with type 1 DM), and the two together in 23 (43.4%) patients. The demographic details are illustrated in Table 1.

The most common site of TDC removal was the right internal jugular vein (77.4%), and in 84.9% of the cases, the TDC was removed as an inpatient procedure. In the majority of the cases (64.2%), the TDC was removed by a noninterventional nephrologist. Fifty-one (96.2%) patients suffered no complications, while only one (1.9%) suffered from minor bleeding which required only prolonged compression time to control bleeding with no further intervention and another one needed TDC removal-related hospitalization (Table 2).

**Figure 1:**
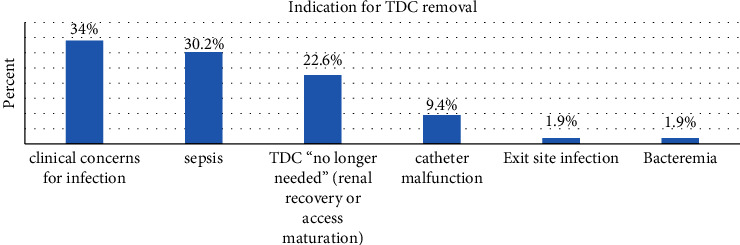
Percent distribution of the reasons for tunnelled hemodialysis catheter removal.

Results show that the most common reasons for TDC removal were sepsis or clinical concerns for infection (64.2%) and TDC “no longer needed” (20.8%) due to recovery of the renal function or access maturation (Figure 1).

There was no significance (*p* > 0.05) between TDC removal and patients' demographics, lab results, chronic conditions, TDC location, place, type of healthcare provider performing TDC removal, and complications (Tables 2 and 3). Blood cultures results were available for 23 patients which revealed that *Staphylococcus aureus* bacteremia was the most frequent in 18 (78%) of them.

## 4. Discussion

Our study revealed that HTN was the most prevalent comorbidity, clearly showing the close association between hypertension and chronic kidney disease (CKD) [7]. In addition, a previous study conducted in Saudi Arabia to evaluate contributing factors for CKD among the family members of patients with hemodialysis revealed that family members of the patients with CKD had a higher prevalence (35.9%) of HTN than those without CKD (29.2%) [8].

The NKF-KDOQI (National Kidney Foundation-Kidney Disease Outcomes Quality Initiative) clinical practice guidelines recommend that the right internal jugular vein (IJV) be used as a preferable access for hemodialysis catheters [9]. The IJV is large, easy to recognize, and has an unimpeded direct path to the right atrium [10]. Consistent with the previous literature and the NKF-KDOQI recommendation, our study found the right IJV to be the most used access point for TDCs.

Catheter use for hemodialysis is widespread despite the National Kidney Foundation and Fistula First Initiative's attempt to reduce its prevalence [11]. TDCs have been associated with numerous complications, such as catheter failure caused by thrombosis or improper positioning and infections associated with catheters [12, 13].

TDC-related infections range from mild skin and soft tissue infection around the exit site to CRB. Antibiotics do not effectively treat CRB and may raise the risk of developing additional complications like endocarditis [14]. Tunnel infection is a catastrophe that can be avoided by guidewire exchange of the TDC, resulting in cure rates similar to TDC removal and replacement [14]. Our study findings also indicate that sepsis or clinical concerns arising from infections (64.2%) are the leading causes of the removal of TDCs. Indications of the removal of TDCs due to catheter-related bacteremia was based on Kidney Disease Outcomes Quality Initiative (KDOQI) recommendations [15]. Moreover, *Staphylococcus aureus* was the most frequent 18 (78%) cause for bacteremia which is in concordance with Lafrance et al. findings in 2008 [16].

Infection and sepsis also imply that these cases need to be treated urgently to prevent complications owing to procedure delays, which could increase morbidity and mortality. The decision of TDCs removal was taken after the failure of all the conservative measurement and failure of antibiotic therapy based on local antibiotic protocol and KDOQI guidelines [15].

TDCs can be removed in different settings, including at the patient's bedside by a nephrologist or in operating rooms by surgeons or interventional radiologists. However, the latter may cause delay in catheter removal due to long wait times for a dedicated operation suite [17]. Nephrologists with the appropriate training can conduct invasive treatments faster and with fewer complications, leading to lower costs for the public health system [18]. A growing body of literature suggests that bedside removal of TDC is a safe and effective procedure regardless of the access site or the reason for removal [5, 17, 18].

According to the research study published in 2013, TDCs were predominantly removed owing to the risk of damage. Bacteremia was observed in 35.4% of the patients, fever in 41.8%, and clinical symptoms of sepsis with hemodynamic instability or respiratory failure. The study's results indicated that TDCs removal by a noninterventional nephrologist was successful with no significant complications [17].

In our study, in the majority of the cases (64.2%), TDC was removed by a noninterventional nephrologist, with a low complication rate (3.8%) in the form of minor bleeding and TDC removal-related hospitalization. One of our patients developed prolonged bleeding which required more observation and monitoring before discharge on the same day with no further intervention. Additionally, another patient required hospitalization due to suspected adherence of the catheter to the vein which required hospitalization and more preparation for catheter removal. Our complication rate was comparable with a previous study which reported a complication rate of *e* 2.8%, mainly comprising of mild bleeding after removal [19]. Moreover, other studies reported prolonged local bleeding in 1.8% the patients [17] or cuff retention in 6.5% [5] of the patients.

### 4.1. Limitations

Our study's limitations include the small sample size and data from a single centre. We wanted to collect data from every hemodialysis centre in Jeddah. However, we could only get approval for KAUH, which unfortunately affected our sample size. Another area for improvement we faced was the need for more information on the brands and manufacturers of the catheters.

## 5. Conclusion

Bedside removal of TDCs by a noninterventional nephrologist is a safe and successful procedure with a low complication rate in most cases. The most common reason for TDC removal is sepsis or clinical concerns arising from infection, which warrants urgent intervention. Bedside removal of the catheters will, therefore, helps prevent delays in treatment in addition to being cost-effective. Also, it helps in reducing the utilization of unnecessary resources and unnecessary radiation exposure due to imaging techniques used by the interventional nephrologist and surgeons to remove TDC.

## Figures and Tables

**Table 1:** Distribution of patients (*n* = 53) according to their demographic characters, lab results, and chronic conditions.

Variables	Number (%)
Age in years	50.94 ± 18.89
BUN (mg/dl)	22.10 (4–56)
Duration of catheter insertion (months)	22 ± 11.7
Creatinine (mg/dl)	770.38 ± 329.532
Platelets count (×10/mm^3^)	197 (65–592)
PTT (sec)	31.70 (22–178)
INR	1.10 (1–4)
WBCs (×10/mm^3^)	8.49 (4–25)
PT (seconds)	12.30 (10–44)
Hemoglobin (g/dl)	8.17 ± 1.66
Sex	
Female	21 (39.6)
Male	32 (60.4)
Underlying medical conditions	
Hypertension	22 (41.5)
Diabetes mellitus, hypertension	19 (35.8)
Diabetes mellitus, hypertension, dyslipidaemia	4 (7.5)
Diabetes mellitus	1 (1.9)
Hypertension, dyslipidaemia	1 (1.9)
Dyslipidaemia	1 (1.9)
No other medical conditions	5 (9.4)

BUN: blood urea nitrogen; PTT: partial thromboplastin time; INR: international normalised ratio; PT: prothrombin time; WBC: white blood cell.

**Table 2:** Relationship between the site of tunnelled hemodialysis catheter (TDC) removal, TDC procedural place, reasons for removal, and complications associated with TDC removal with the type of healthcare provider performing the procedure.

Variables	TDC removal			*χ* ^2^	*p* value
	Interventional radiologist/nephrologist no. (%) 13 (24.5%)	Noninterventional nephrologist no. (%) 34 (64.2%)	Surgeon no. (%) 6 (11.3%)		
Location of TDC removed					
Left femoral vein	0 (0.0)	2 (100)	0 (0.0)	3.46	0.748
Left internal jugular vein	1 (12.5)	6 (75)	1 (12.5)		
Right femoral vein	0 (0.0)	2 (100)	0 (0.0)		
Right internal jugular vein	12 (29.3)	24 (58.5)	5 (12.2)		
Place of TDC removal					
Inpatient	13 (28.9)	26 (57.8)	6 (13.3)	5.26	0.072
Outpatient	0 (0.0)	8 (100)	0 (0.0)		
Indication for TDC removal					
Exit site infection	0 (0.0)	1 (100)	0 (0.0)		
Catheter malfunction	1 (20%)	3 (60%)	1 (20%)		
Bacteremia	0 (0.0)	1 (100)	0 (0.0)	4.42	0.810
Clinical concerns for infection	6 (33.3%)	10 (55.6%)	2 (11.1%)		
Sepsis	4 (25%)	9 (56.3%)	3 (18.8%)		
TDC “no longer needed” (renal recovery or access maturation)	2 (16.7%)	10 (83.3%)	0 (0.0)		
Complication of TDC removal					
Bleeding	0 (0.0)	1 (100)	0 (0.0)		
Hospitalization related to catheter removal	0 (0.0)	0 (0.0)	1 (100)	8.51	0.074
None	13 (25.5)	33 (64.7)	5 (9.8)		

TDC: tunnelled hemodialysis catheter.

**Table 3:** Relationship between the tunnelled hemodialysis catheter removal procedure and patients' demographics, lab results, and underlying medical conditions.

Variables	TDC removal			*χ* ^2^	*p* value
	Intervention radiologist/nephrologist no. (%) 13 (24.5%)	Noninterventional nephrologist no. (%) 34 (64.2%)	Surgeon no. (%) 6 (11.3%)		
Age	52.46 ± 22.55	51.88 ± 17.77	42.33 ± 17.46	0.69^*∗*^	0.502
BUN (mg/dl)	22.60 (11–52)	21.65 (4–56)	22.40 (14–41)	2^*∗∗*^	0.566
Creatinine (mg/dl)	716.15 ± 320.22	763.56 ± 336.16	926.5 ± 316.511	0.85^*∗*^	0.433
Platelets count (×10/mm^3^)	163 (66–271)	205.5 (65–592)	192 (81–225)	2^*∗∗*^	0.37
PTT (sec)	31.10 (26–119)	31.85 (22–178)	32.35 (28–42)	2^*∗∗*^	0.984
INR	1.10 (1–3)	1.10 (1–4)	1.12 (1–3)	2^*∗∗*^	0.956
WBCs (×10/mm^3^)	7.92 (4–20)	8.42 (4–25)	10.20 (8–19)	2^*∗∗*^	0.346
1PT	13 (11–17)	12.20 (10–44)	12.60 (11–16)	2.1^*∗∗*^	0.949
Hemoglobin (g/dl)	8.68 ± 1.46	8.01 ± 1.78	7.98 ± 1.36	0.8^*∗*^	0.453
Duration of catheter insertion (months)	21 ± 15	19 ± 9.7	24 ± 10.7	2.4^*∗∗*^	0.171
Gender					
Female	4 (19)	14 (66.7)	3 (14.3)	0.73	90.694
Male	9 (28.1)	20 (62.5)	3 (9.4)		
Underlying medical conditions					
Hypertension	5 (22.7)	14 (63.6)	3 (13.6)		
Diabetes mellitus, hypertension	5 (25.3)	13 (68.4)	1 (5.3)		
Diabetes mellitus, hypertension, dyslipidaemia	1 (25)	2 (50)	1 (25)	6.19	0.906
Diabetes mellitus	0 (0.0)	1 (100)	0 (0.0)		
Dyslipidaemia	1 (100)	0 (0.0)	0 (0.0)		
Hypertension, dyslipidaemia	0 (0.0)	1 (100)	0 (0.0)		
None	1 (20)	3 (60)	1 (20)		

^*∗*^ = one-way ANOVA test; ^*∗∗*^ = Kruskal–Wallis test. TDC: tunnelled hemodialysis catheters; BUN: blood urea nitrogen; PTT: partial thromboplastin time; INR: international normalised ratio; PT: prothrombin time; WBC: white blood cell.

## Data Availability

The data used for the findings in this study are available on request from the corresponding author.

## References

[1] Schwab S. J., Beathard G. (1999). The hemodialysis catheter conundrum: hate living with them, but can’t live without them. *Kidney International*.

[2] Fülöp T., Tapolyai M. B., Agarwal M., Lopez-Ruiz A., Molnar M. Z., Dossabhoy N. R. (Sep. 2017). Bedside tunneled dialysis catheter removal-A lesson learned from nephrology trainees. *Artificial Organs*.

[3] Clark E., Kappel J., MacRae J. (2016). Practical aspects of nontunneled and tunneled hemodialysis catheters. *Canadian Journal of Kidney Health and Disease*.

[4] Alomari A. I., Falk A. (2007). The natural history of tunneled hemodialysis catheters removed or exchanged: a single-institution experience. *Journal of Vascular and Interventional Radiology*.

[5] Fülöp T., Rodriguez B., Kosztaczky B. A. (2015). Tunneled hemodialysis catheter removals by non-interventional nephrologists: the university of Mississippi experience. *Seminars in Dialysis*.

[6] Al-Sayyari A. A., Shaheen F. A. (2011). End stage chronic kidney disease in Saudi Arabia. A rapidly changing scene. *Saudi Medical Journal*.

[7] Agarwal R., Peixoto A. J., Santos S. F. F., Zoccali C. (2009). Out-of-office blood pressure monitoring in chronic kidney disease. *Blood Pressure Monitoring*.

[8] Mousa D., Alharbi A., Helal I. (2021). Prevalence and associated factors of chronic kidney disease among relatives of hemodialysis patients in Saudi Arabia. *Kidney International Reports*.

[9] Vascular Access Work Group (2006). Clinical practice guidelines for vascular access. *American Journal of Kidney Diseases*.

[10] Bannon M. P., Heller S. F., Rivera M. (2011). Anatomic considerations for central venous cannulation. *Risk Management and Healthcare Policy*.

[11] Banerjee S. (2009). Dialysis catheters and their common complications: an update. *The Scientific World Journal*.

[12] Beathard G. A., Litchfield T. (2004). Effectiveness and safety of dialysis vascular access procedures performed by interventional nephrologists. *Kidney International*.

[13] Cetinkaya R., Odabas A. R., Unlu Y., Selcuk Y., Ates A., Ceviz M. (2003). Using cuffed and tunnelled central venous catheters as permanent vascular access for hemodialysis: a prospective study. *Renal Failure*.

[14] Saad T. F. (2001). Central venous dialysis catheters: catheter-associated infection. *Seminars in Dialysis*.

[15] Lok C. E., Huber T. S., Lee T. (2020). KDOQI clinical practice guideline for vascular access: 2019 update. *American Journal of Kidney Diseases*.

[16] Lafrance J. P., Rahme E., Lelorier J., Iqbal S. (2008). Vascular access–related infections: definitions, incidence rates, and risk factors. *American Journal of Kidney Diseases*.

[17] Fülöp T., Tapolyai M., Qureshi N. A. (2013). The safety and efficacy of bedside removal of tunneled hemodialysis catheters by nephrology trainees. *Renal Failure*.

[18] Quintiliano A., Praxedes M. R. G. (2020). Effectiveness, safety and cost reduction of long-term tunneled central venous catheter insertion in outpatients performed by an interventional nephrologist. *Brazilian Journal of Nephrology*.

[19] Dossabhoy N. R., Sangha B., Tapolyai M. B., Fülöp T. (2016). Outpatient removal of tunneled dialysis catheters by nephrology fellows in training at a veterans affairs medical center. *The Journal of Vascular Access*.

